# Human Adipose Stromal Cells (ASC) for the Regeneration of Injured Cartilage Display Genetic Stability after *In Vitro* Culture Expansion

**DOI:** 10.1371/journal.pone.0077895

**Published:** 2013-10-28

**Authors:** Simona Neri, Philippe Bourin, Julie-Anne Peyrafitte, Luca Cattini, Andrea Facchini, Erminia Mariani

**Affiliations:** 1 Laboratory of Immunorheumatology and Tissue Regeneration/RAMSES, Rizzoli Orthopedic Institute, Bologna, Italy; 2 Etablissement Français du Sang Pyrénées Méditerranée (EFS-PM), Toulouse, France; 3 CSA21, Toulouse, France; 4 STROMALAB, UMR 5273 Centre national de la Recherche Scientifique (CNRS)/Université Paul Sabatier, U1031 Institut National de la Santé et de la Recherche Médicale (INSERM), Toulouse, France; 5 Medical and Surgical Sciences Department, University of Bologna, Bologna, Italy; Northwestern University, United States of America

## Abstract

Mesenchymal stromal cells are emerging as an extremely promising therapeutic agent for tissue regeneration due to their multi-potency, immune-modulation and secretome activities, but safety remains one of the main concerns, particularly when *in vitro* manipulation, such as cell expansion, is performed before clinical application. Indeed, it is well documented that *in vitro* expansion reduces replicative potential and some multi-potency and promotes cell senescence. Furthermore, during *in vitro* aging there is a decrease in DNA synthesis and repair efficiency thus leading to DNA damage accumulation and possibly inducing genomic instability. The European Research Project ADIPOA aims at validating an innovative cell-based therapy where autologous adipose stromal cells (ASCs) are injected in the diseased articulation to activate regeneration of the cartilage. The primary objective of this paper was to assess the safety of cultured ASCs. The maintenance of genetic integrity was evaluated during *in vitro* culture by karyotype and microsatellite instability analysis. In addition, RT-PCR array-based evaluation of the expression of genes related to DNA damage signaling pathways was performed. Finally, the senescence and replicative potential of cultured cells was evaluated by telomere length and telomerase activity assessment, whereas anchorage-independent clone development was tested *in vitro* by soft agar growth. We found that cultured ASCs do not show genetic alterations and replicative senescence during the period of observation, nor anchorage-independent growth, supporting an argument for the safety of ASCs for clinical use.

## Introduction

Mesenchymal Stromal Cells (MSCs) are a population of adherent cells that are able to differentiate into mesenchymal lineages (cartilage, bone and fat tissue), as well as into other types of lineages not derived from the mesodermal layer [1].

MSCs obtained from bone marrow (BM) represent a standard in the field of adult stem cell biology and clinical applications; however, stromal cells obtained from adipose tissue are becoming an attractive alternative due to their minimally invasive accessibility and plentiful availability.

Adipose tissue originates from mesodermal germ layer and it is the most shared connective tissue in humans. It is mainly distributed as subcutaneous and visceral fat [2] and its removal (generally by liposuction) supplies a large amount of mesenchymal stem cells, for regenerative medicine purposes (about 300,000/ml) [3], [4].

The enzymatic digestion of adipose tissue releases a heterogeneous mix of cells, the stromal vascular fraction (SVF), which contains the stromal population of adipose derived stromal cells [5]. The culture of the crude SVF allows the expansion of a relatively homogeneous fibroblastic-like population, adherent to plastic, with a wide proliferative capacity and a stromal surface phenotype [6], [7], potentially differentiating in multiple lineages [8]–[12]. This population is identified as adipose-derived stromal/stem cells (ASCs), according to IFATS’s recommendations [13], [14]. ASCs share a lot of similarities, such as morphology, distribution of surface antigens and multi-potency with other stem cells obtained, for example, from BM or umbilical cord blood [15].

The European Regulation defines the use of mesenchymal stem cells (either derived from BM or from adipose tissue) as advanced therapy medicinal products (DIRECTIVE 2001/83/EC of the European Parliament and of the Council of 6 November 2001 on the Community Code Relating to Medicinal Products for Human Use. Available from URL: http://www.emea.europa.eu/pdfs/human/pmf/2001-83-EC.pdf; Regulation (EC) no 1394/2007 of the European Parliament and of the Council. Available from URL: http://eur-lex.europa.eu/LexUriServ/LexUriServ).

The production of clinical-grade MSCs in agreement with GMP procedures requires the careful identification and control of all the phases of cell manipulation and release. In addition to bacteriological tests, that attest the sterility of the cell product, the methods of expansion must assure and maintain the phenotypic and functional characteristics of the cells as well as their genomic stability during the *in vitro* culture period [16]–[19].

The spontaneous transformation of human mesenchymal stem cells during *in vitro* expansion remains a major safety problem [1], [17]–[18], [20]. Several studies pointed to the safety of cultured human mesenchymal stem cells by molecular evaluation of genomic stability [21]–[22] and by evaluating tumorigenicity *in vivo* [23]–[28]. It is demonstrated that *in vitro*-expanded human MSC acquire chromosomal aberrations [23], [29]–[33] and there is still an open debate about the relevance of these alterations. Some authors argue the irrelevance of these modifications and their transient nature [30], [33]–[34], whereas others suggest particular caution when interpreting these findings [31], [35]. However, at present very few data were provided about human MSC transformation in culture [36] and two impressive studies describing malignant transformation were subsequently retracted by the authors [37]–[40].

The aim of this study was to evaluate the potential susceptibility of *in vitro* expanded ASC to genetic alterations at different *in vitro* culture time points due to the effect of *in vitro* ageing, replicative stress and culture conditions to define appropriate quality controls and release criteria for the clinical use of these cells. ASC isolation and culture were performed according to GMP and in agreement with the UE ADIPOA project, which involves the present authors and concerning an innovative cell-based therapy for cartilage regeneration in knee osteoarthritis by ASC injection in the diseased articulation.

To impose extreme conditions on cells, longer observation times were chosen intentionally compared with those defined for releasing cells for clinical applications (i.e. the end of P1, corresponding to 14 days of culture).

To evaluate genetic integrity of *in vitro*-expanded ASCs, DNA damage accumulation was analyzed both at chromosome and molecular levels, by karyotype and MicroSatellite Instability (MSI) analysis, respectively. Karyotyping is the gold standard to evaluate gross DNA alterations, but possible small molecular changes are not detected by this technique. The MisMatch Repair (MMR) System is the main post-replicative correction pathway whose efficiency can be monitored by MSI analysis. The rationale to evaluate this repair system lies in its fundamental role for genomic stability maintenance [41]–[43] in actively replicating cells such as those being studied. In addition, both oxidative stress (known to be induced by culture conditions) [44]–[46] and *in vitro*/*in vivo* aging [47]–[50] were shown to be capable of altering MMR system efficiency, thus leading to genetic instability. Therefore, MSI analysis might be used as a possible biosafety marker of long-term culture genetic stability at molecular level. Besides these investigations, RT-PCR array-based evaluation of the expression of genes related to DNA damage and repair was performed.

Finally, the senescence and replicative potential of cultured cells was evaluated by telomere length and telomerase activity assessment, whereas anchorage-independent clone development was tested *in vitro* by soft agar growth.

The present results argue in favor of the safety of ASC-cultured cells for clinical applications.

## Materials and Methods

### Ethics Statement

Research was performed in the context of the AdipoA EU FP7 project, under the approval of AFSSAPS (Agence française de sécurité sanitaire des produits de santé), reference n. TC301 of December 28, 2011, with donor written informed consent. Patient names were replaced by arbitrary codes to remain anonymous.

### ASC Isolation and Culture

Clinical grade ASCs were isolated from 6 donor lipoaspirates (A–F) according to Good Manufacturing Practice (GMP) as described [51]. Informed consent was obtained from all donors. Briefly, the adipose tissue (30±1 g) was harvested by liposuction under local anesthesia. All donors (1 woman and 5 men, mean age ± SD: 66±20 years, mean Body Mass Index ± SD: 26±2.1 Kg/m^2^) gave their informed consent to the study. Fat was digested with collagenase NB6 GMP-grade from *C. histolyticum* (Serva Elecrophoresis, Germany) at 37°C for 45 minutes and centrifuged (600×g for 10 min). The SVF was seeded into a CellStack™ (Corning, France) chamber at the density of 4×10^3^ cell/cm^2^ in α-MEM supplemented with platelet lysate (PLP) (EFS-PM own production). On day 8, the cells were trypsinized and expanded at 2×10^3^ cells/cm^2^ until day 14 (end of passage 1). Cell counting and viability were performed using trypan blue exclusion dye on day 8 and 14. The mean number of cells recovered after 14 days was 2.3×10^8^±0.5×10^8^. ASCs were then cultured until they reached at least 25 population doublings and harvested at different times of *in vitro* expansion, counted and liquid-nitrogen frozen. A total of 6 donors and 36 passages were analyzed (see Table 1 for details). All molecular analyses were performed on thawed ASCs. When the amount of recovered cells was low, only selected analyses were performed.

**Table 1:** Characteristics of ASC analyzed at different culture passages.

	Passages [days of culture]										
Samples	0	1	2	3	4	5	6	7	9	14	
A	7.9 [8]	12.8 [14]	17.2 [21]	21.2 [28]	26.0 [35]	30.2 [42]	34.9 [49]	n.d.	45.7 [70]	60.1 [120]	
B	n.d.	11.2 [14]	14.8 [21]	19.2 [28]	23.5 [35]	27.3 [42]	31.0 [49]	n.d.	n.d.	n.d.	
C	7.8 [8]	13.0 [15]	17.7 [23]	n.d.	25.4 [36]	29.8 [43]	33.3 [50]	n.d.	n.d.	n.d.	
D	n.d.	10.6 [14]	n.d.	16.2 [28]	n.d.	n.d.	22.2 [62]	24.8 [96]	n.d.	n.d.	
E	8.1 [8]	12.9 [14]	n.d.	21.1 [28]	24.8 [35]	28.6 [42]	31.9 [49]	n.d.	n.d.	n.d.	
F	6.4 [8]	10.7 [14]	15.3 [20]	n.d.	n.d.	28.7 [40]	32.7 [47]	n.d.	n.d.	n.d.	
Mean±SD	7.6±0.8	11.9±1.1	16.2±1.4	19.4±2.3	24.9±1.1	28.9±1.2	31.0±4.5				

Cumulative Population Doublings and [days of culture] of all the samples at the different culture passages analyzed are reported.

### ASCs Phenotypical Analysis

The phenotype was carried out by flow cytometric analysis for the CD markers CD14, CD34, CD45, CD73, CD90 (BD Pharmingen, San José, USA) according to a previously described method [52]. Briefly, at the first passage, 1×10^5^ ASCs were suspended in flow cytometry buffer (FCB) and then stained with a saturating amount of conjugated antibodies or their respective controls. The cells were analyzed using an EPICS XL flow cytometer (Beckman-Coulter, USA).

### Replication Rate

Population doubling (PD) was calculated at each culture passage, based on recovered vs. seeded cells from the cell count by using the equation NH/NI = 2^PD^, where NH = number of harvested cells and NI = number of seeded cells [53]. The PD calculated at each passage was then added to the previous PD level, to yield the cumulative population doubling (CPD) level. For the first cell passage, the PDs were calculated in relationship to the CFU-F number in the SVF (P0), according to the following formula: NH/(NSVF*CF) where NSVF = number of seeded SVF cells and CF = CFU-F frequency in the SVF population.

### Karyotype Analysis

Cytogenetic evaluation was performed by the Laboratoire de cytogénétique of the EFS-PM. Thirty metaphases per sample were evaluated, at selected culture passages, by standard G-banding karyotype following European guidelines (General Guidelines and Quality Assurance for Cytogenetics, European Cytogeneticists Association, http://www.e-c-a.eu).

### MicroSatellite Instability (MSI) Analysis

Total DNA from ASC pellets (100,000 cells) was extracted with the QIAamp DNA Mini Kit (Qiagen GmbH, Germany), spectrophotometrically quantified and amplified by PCR at five different microsatellite sequences (CD4, VWA, Fes, Tpox and P53) as described [54]. PCR products were run on a 10% polyacrylamide non-denaturing gel together with ladders and visualized by Sybr green staining. Genetic typing of each sample was performed by identification of the alleles at each locus by comparison with ladders made up from a mixture of known alleles. The allelic patterns were checked for MSI at different *in vitro* passages (appearance of allele shifts or additional bands) of each ASC preparation.

### Array-based Gene Expression Analysis

ASC samples from donors displaying a normal karyotype (A, B, C, E, F) were evaluated at P1 and P5), whereas donor D, showing karyotype abnormalities, was tested at each available culture passage (P1, P3, P6 and P7). In addition, donor sample A was also tested at P14, the most advanced culture passage available. Total cellular RNA was isolated from the cell pellets (200,000 cells) with RNA pure isolation reagent (Euroclone, Italy), treated with the RNase-Free DNase Set (Qiagen, Germany) and cleaned-up in RNeasy mini columns (Qiagen) following manufacturer’s instructions, to avoid possibly contaminating DNA. Quantity and quality of the total RNA for each preparation was determined using a Nanodrop 2000 spectrophotometer (Thermo Scientific, Wilmington, DE, USA). Total RNA from each sample (0.8 µg) was reverse transcribed by the RT^2^ First strand kit (Qiagen). Expression of a focused panel of 84 genes involved in human DNA Damage Signaling Pathways together with genomic DNA control, reverse-transcription control, positive and negative control and five housekeeping genes (Beta-2-microglobulin, Hypoxanthine phosphoribosyltransferase 1, Ribosomal protein L13a, Glyceraldehyde-3-phosphate dehydrogenase, beta-actin) was evaluated by the RT^2^ Profiler PCR Array PAHS-029 (Qiagen). Amplification was performed with the RT^2^ SYBR Green ROX Fast Mastermix (Qiagen) in a Rotor-gene 6000 real-time analyzer (Corbett, Concorde, NSW, Australia). The threshold cycle (Ct) values for all the genes were calculated and considered as a negative call when ≥33. Relative gene expressions were calculated by using the ΔΔCt method relative to the housekeeping genes showing Ct value consistency. Results were analyzed by a Web-based PCR Array Data Analysis Software available at http://pcrdataanalysis.sabiosciences.com/pcr/arrayanalysis.php. by comparing samples at different culture passages and by comparing samples with normal karyotype to those displaying aneuploidies.

### Flow-FISH Determination of Telomere Length

The cells were treated as already described [55]. Briefly, 150,000 ASCs were washed and resuspended in hybridization buffer (70% deionized formamide, 20 mM Tris buffer pH 7.0, 1%BSA) containing 2.0 µM FITC labeled (CCCTAA)_3_ peptide nucleic acid (PNA) probe (Biosynthesis INC, USA), specific for the telomeric sequence. ASC samples incubated in hybridization mixture without probe were used as negative controls. Samples and controls were heat-denatured for 7 min at 80°C and left to hybridize 2 hours at room temperature in the dark. The cells were then washed and analyzed by flow cytometry (FACSCanto II equipped with a 488 nm laser; FITC emission collected through 525/40BP; 5000 events acquired). Telomere fluorescence was evaluated and expressed as the difference between the geometric mean fluorescence intensity (MFI) of cells hybridized with telomere PNA-probe and the corresponding unstained control sample.

A direct proportionality between mean telomere length and fluorescence intensity was previously demonstrated in human lymphocytes by parallel determination of mean telomere length by Southern blotting (Pearson’s correlation: r = 0.737; p = 0.0001, unpublished results by Neri S. et al.).

### PCR-Elisa Determination of Telomerase Activity

Quantitative determination of telomerase activity was performed using the TeloTAGGG telomerase PCR Elisa Kit (ROCHE, Germany), a photometric enzyme immunoassay, following the manufacturer’s instructions. Briefly, extracts were prepared from 2×10^5^ ASCs from each sample and divided into two aliquots: one was used as the negative control after heat inactivation of telomerase at 80°C for 30 min, the other was used to evaluate the telomerase-mediated addition of telomeric sequences. The products were amplified by PCR and, to exclude false negative results, a 216 bp length internal standard was simultaneously amplified. The amounts of hexamers added by the telomerase contained in each lysate were then evaluated by the Elisa technique using TTAGGG specific probes. Relative telomerase activity (RTA) was calculated for each sample in comparison with an internal control showing low telomerase activity (low control = 10^−6^ pmoles of template DNA corresponding to a product with eight telomeric repeats), after subtraction of the negative corresponding sample with heat inactivated telomerase [absorbance reading (As): 450 nm; reference wavelength: 690 nm]. The following formula was used: %RTA = [(As sample−As heat-treated sample)/As internal standard]/[(As control template−As lysis buffer)/As internal standard]×100.

The BCR-ABL positive tumor cell line K562 with activated telomerase [56] was used to compare ASC telomerase activity with a positive control.

### Soft Agar Growth

For *in vitro* testing of anchorage-independent colony development, the ASCs of four cases and six passages, namely A (P14), B (P6), D (P3, P7) F (P0, P2), were cultured in soft agar. The cells (30,000 per sample) were resuspended in 0.3% Agarose (Sigma, USA), final concentration, in D-MEM and 10% FCS and stratified on a bottom layer of 0.5% pre-solidified Agarose in six-well plates. Plating was performed in duplicate and 1 ml of D-MEM complete medium was carefully added on the top of each well to ensure nutritive supplies and to prevent drying. The medium was changed every two days. Plates were incubated at 37°C and 5% CO_2_ and evaluated for the presence of colonies at days 20 and 30. The Saos-2 osteosarcoma cell line, simultaneously cultured in a separate plate, was used as positive control. A well containing only the two layers of Agarose and medium was included in each plate and used as negative control.

### Statistical Analysis

Results are expressed as mean±SD (standard deviation) or ±SEM (standard error mean, for phenotype analysis), median and interquartile ranges, as appropriate. Comparisons among passages were performed by the Friedman Anova test and between passages by the Wilcoxon matched pairs test.

Statistical analysis was performed using the Statistica 6 package for Windows (StatSoft, USA).

Non-supervised hierarchical cluster analysis was used to compare ASC samples for their expression of DNA damage signaling genes by a SABiosciences Software (Qiagen) available at http://pcrdataanalysis.sabiosciences.com/pcr/arrayanalysis.php.

## Results

### ASC Growth in Culture

Cells were grown as monolayers for several periods, and then the cultures were harvested to allow data analysis. All cultures showed fibroblastic-like cells growing attached to the plastic. As expected, the cells progressively adopted an enlarged phenotype when they approached senescence. Culture passages analyzed for the six donors together with the corresponding CPD and days of culture are represented in Table 1.

The average number of CPD at the end of P1 (scheduled time for injection, corresponding to 14 days of culture) was 11.9±1.14 and 31.0±4.53 at P6 (corresponding to 51.0±5.48 days). Sample A was analyzed until P14 corresponding to 60.1 CPD and 120 days of culture.

**Figure 1:**
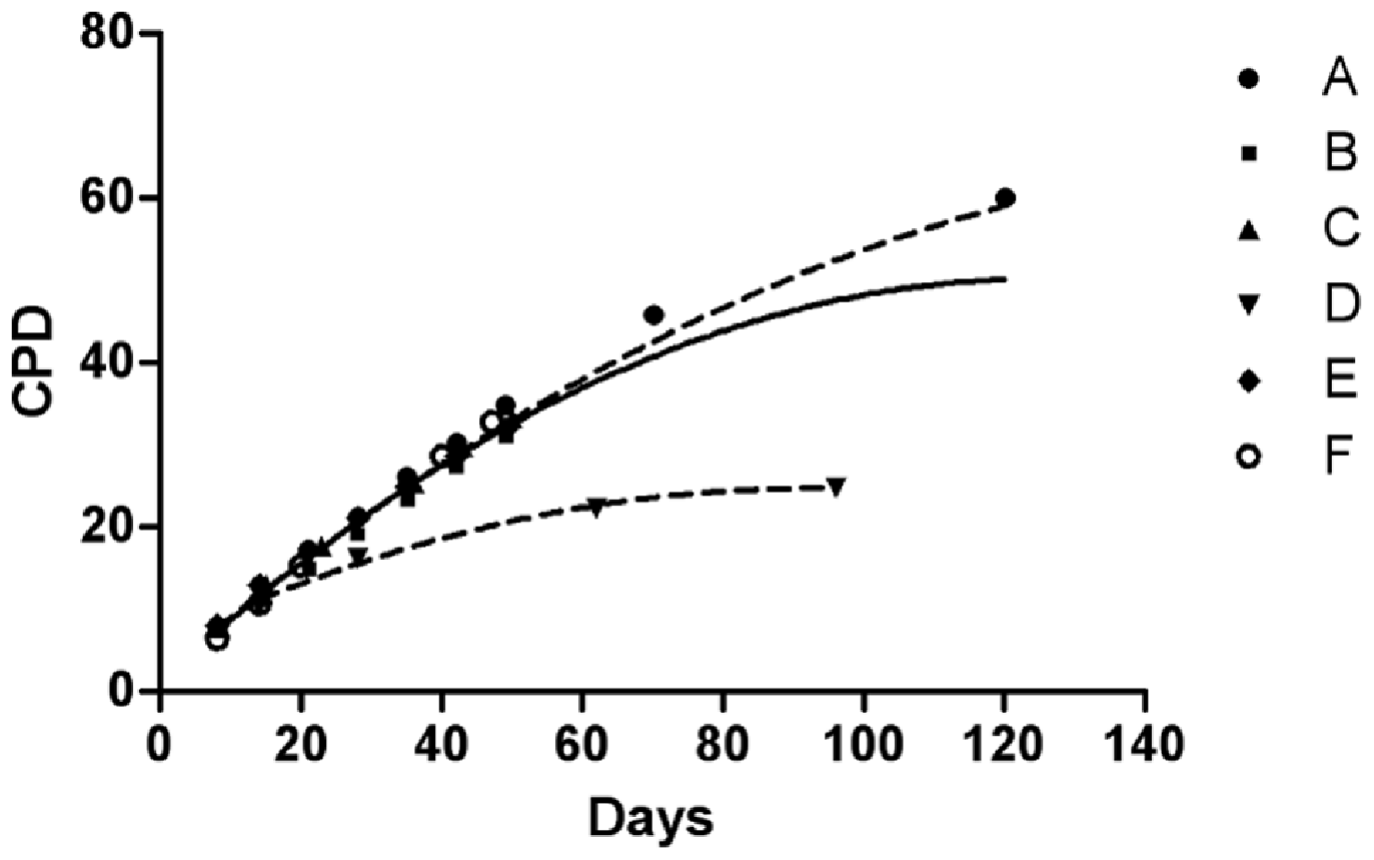
Growth curves. The continuous line represents the regression fit of all the samples. The upper dashed line represents the regression fit of samples excluded sample D. The lower dashed line indicates the regression fit of sample D alone.

Growth curves (Fig. 1) show a low variability in the proliferative capacity among donors in terms of CPD accomplished in the same time interval, except for donor D, showing a consistent decrease of population doublings starting from passage 3, about 15% lower than the minimum value observed in the passage among samples and reaching almost 30% decrease at passage 6.

The relationship between CPD and days of culture of all the analyzed samples is described by a quadratic equation (CPD = 3.3639+0.6663*days −0.0024*days^2^).

The proliferation rate (CPD number with respect to days of culture) in the six donor samples progressively decreased with time, as expected: from 0.947±0.096 at P0 to 0.837±0.073 at release time (P1) to 0.692±0.028 at P5 (p = 0.043 by Wilcoxon’s matched pairs test, P1 vs. P5).

### Immunophenotype Analysis

ASCs had a classical phenotype at the end of the first passage. They did not express CD14 or CD45 (1.9±0.7% and 0.22±0.1%, respectively), they had a very low expression of CD34 (0.6±0.2%) and they all expressed CD73 and CD90 (99.7±0.2% and 99.8±0.2%, respectively).

### Cytogenetic Stability

**Figure 2:**
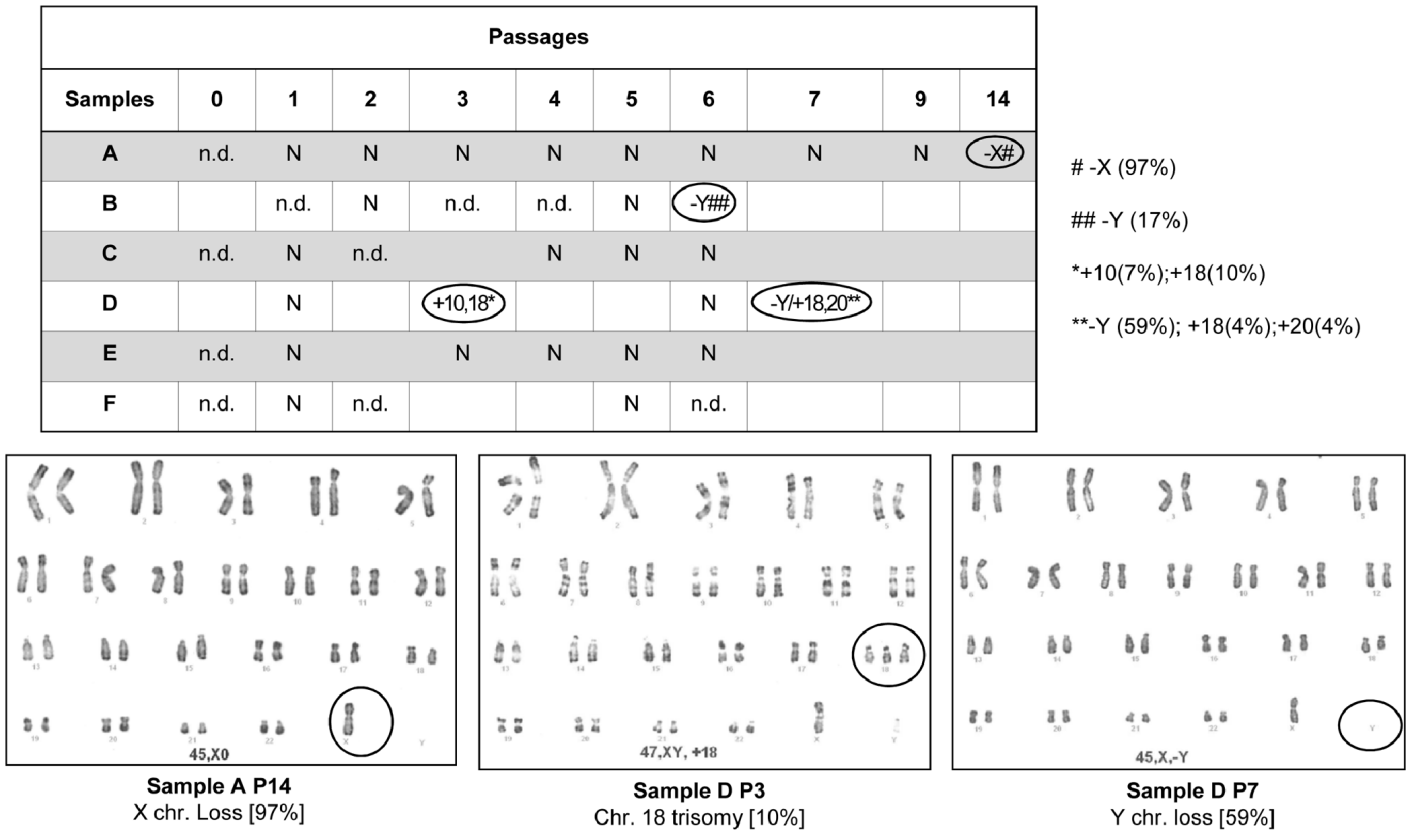
Karyotype. Data are summarized in the table (above) and refer to 30 analyzed mitoses for each sample. Three abnormal karyotypes are shown (below). N = normal karyotype; n.d. = not determined.

None of the analyzed samples showed karyotype alterations until the second culture passage. Three out of six donor samples (C, E, F) showed no karyotype alterations throughout the culture period, whereas two donor samples (A and B) showed a sex chromosome loss at advanced culture as the sole anomaly (Fig. 2). Specifically, sample A lost the X chromosome at P14 in 97% of metaphase cells, whereas sample B showed a Y chromosome loss at P6 in 17% of metaphase cells. Only one donor (sample D) presented autosomal aneuploidies, which were already present at P3. In particular, chromosome 10 and 18 trisomies in 7% and 10% of metaphase cells were respectively detected at P3, whereas chromosome 18 and 20 trisomies in 4% of metaphase cells were detected at P7, together with a Y chromosome loss in 63% of metaphase cells (Fig. 2).

### Microsatellite Instability Analysis

**Figure 3:**
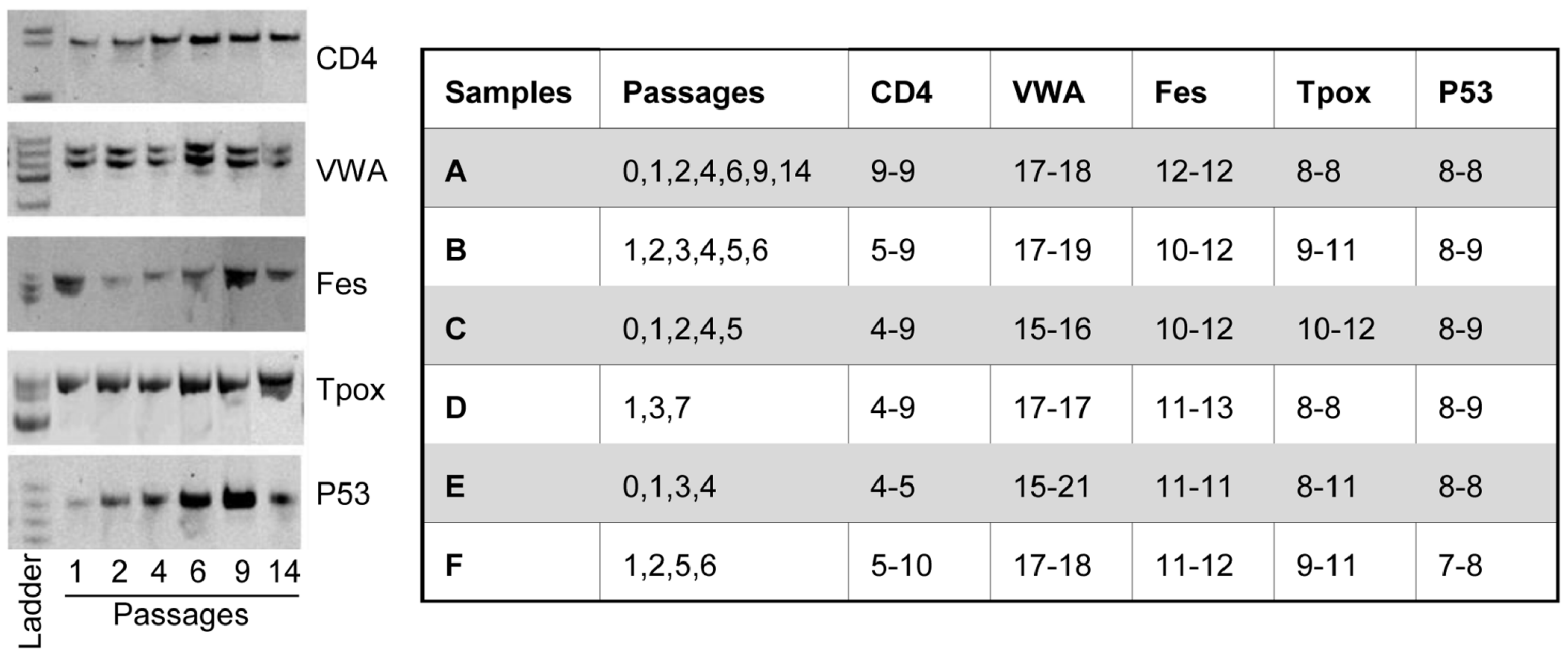
Microsatellite instability (MSI) analysis. On the left: MSI analysis of sample A is shown as an example. Allele patterns for each of the five analyzed microsatellite sequences (CD4, VWA, Fes, Tpox and P53) were identified by acrylamide gel separation and compared among culture passages (P1, 2, 4, 6, 9 and 14). Each allele pattern appears to be stable throughout the culture period. On the right: Table summarizing MSI analysis of the 6 ASC samples. Allele nomenclature refers to the number of repeats [47]. For each ASC sample, allele patterns for the five analyzed microsatellite loci (CD4, VWA, Fes, Tpox and P53) were maintained at all the different analyzed culture passages.

Allele typing for the six donors at five repeated sequences is indicated in Fig. 3, as resulting from the analysis performed at different culture passages. No cases of microsatellite instability were observed and allele patterns were maintained throughout the culture period for all the analyzed donors, thus showing that, in this cellular culture model, repeated replications *in vitro* did not alter genetic stability at simple sequence repeats.

### DNA Damage Signaling Pathway Expression Profile

**Figure 4:**
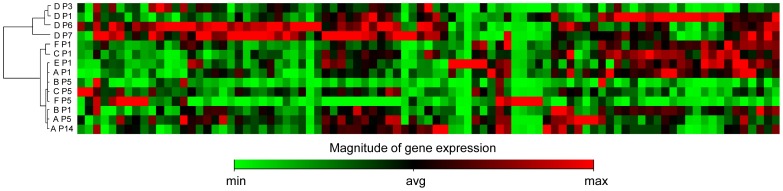
Gene expression analysis. Dendrogram obtained by hierarchical clustering of 6 ASC samples (A–F) at different culture passages (P, from 1 to 14) based on the expression of 84 genes involved in DNA damage signaling pathways. Gene expression levels are shown as a heat map for each gene.

To further explore cell response to repeated replications *in vitro*, we investigated the gene expression patterns of DNA damage signaling pathways by RT-PCR arrays. All analyzed ASC samples were evaluated by non-supervised hierarchical cluster analysis, grouping samples according to how closely related their gene expression profiles were (Fig. 4).

Interestingly, two main clusters were highlighted, one containing all samples, except sample D, at all analyzed passages, and the other containing sample D at the four different passages analyzed (P1, P3, P6 and P7). In particular, the finding of all sample D passages in a separate cluster, suggests a general different behavior for this donor sample that seems to be independent of culture passages. Coincidently, sample D was the only one showing a consistent slowdown of the population doublings starting from initial culture passages (Fig. 1) and the only one displaying autosomal chromosome alterations during culture (Fig. 2).

Semi-quantitative gene expression analysis was focused on highly responsive genes: a 2-fold change threshold for up- and down-regulation was applied (p≤0.05). To assess the effect of *in vitro* culture time on DNA damage-related gene expression, we compared samples at P1 with those at P5 and the only available sample at P14. We observed very few genes whose expression was significantly modified between P1 and P5 (Table 2), notably, all significantly modified genes showed a reduced expression, thus indicating a general down-regulation due to culture time.

**Table 2:** Effect of culture expansion on DNA damage signaling pathway gene expression.

			P1 vs P5		P1 vs P14	
Symbol	Accession	Description	P value	Fold changes	P value	Fold changes
BRCA1	NM_007294	Breast cancer 1	0.0002	−8.82	n.a.	−7.36
DMC1	NM_007068	Dosage suppressor of mck1 homolog, meiosis-specific homologous recombination	0.0008	−11.11	n.a.	−29.38
EXO1	NM_130398	Exonuclease I	n.s.	−4.24	n.a.	−16.05
FEN1	NM_004111	Flap structure-specific endonuclease I	0.0015	−4.93	= = =	= = =
GML	NM_002066	Glycosylphosphatidylinositol anchored molecule like protein	= = =	= = =	n.a.	−4.35
GTSE1	NM_016426	G2 and S-phase expressed I	0.0103	−17.09	n.a.	−22.61
IP6K3	NM_054111	Inositol hexakiphosphate kinase 3	= = =	= = =	n.a.	24.07
XRCC2	NM_005431	X-ray repair complementing defective repair in Chinese hamster cells 2	= = =	= = =	n.a.	−7.02

Results from RT-PCR array analysis are shown as fold changes between P1 (n = 6) and P5 (n = 4) or P14 (n = 1).

n.a. = not applicable; n.s. = not significant.

This behavior was even more evident at P14, even if the availability of only one sample at this passage did not allow statistical analysis to be performed (Table 2).

Whereas sample D at the beginning of the culture was indistinguishable from the other ones, culture time induced an up-regulation of some genes not observed in samples with normal karyotype.

In particular, sample D at P7 showed an up-regulation of BTG2 (B cell Translocation Gene family member 2, NM_006763; 4.50 folds), GADD45G (Growth arrest and DNA-damage-inducible, gamma, NM_006705; 4.18 folds), IP6K3 (Inositol hexakiphosphate kinase 3, NM_054111; 15.45 folds), MAP2K6 (Mitogen-activated protein kinase kinase 6, NM_002758; 9.61 folds) genes compared to samples with normal karyotype at P5.

In agreement with the absence of microsatellite instability, MMR gene expression (MLH1, MSH2, PMS1, PMS2, MLH3 and PCNA), as evaluated by array analysis, showed no significant modification until very advanced culture passages.

### Telomere Length and Telomerase Activity

**Figure 5:**
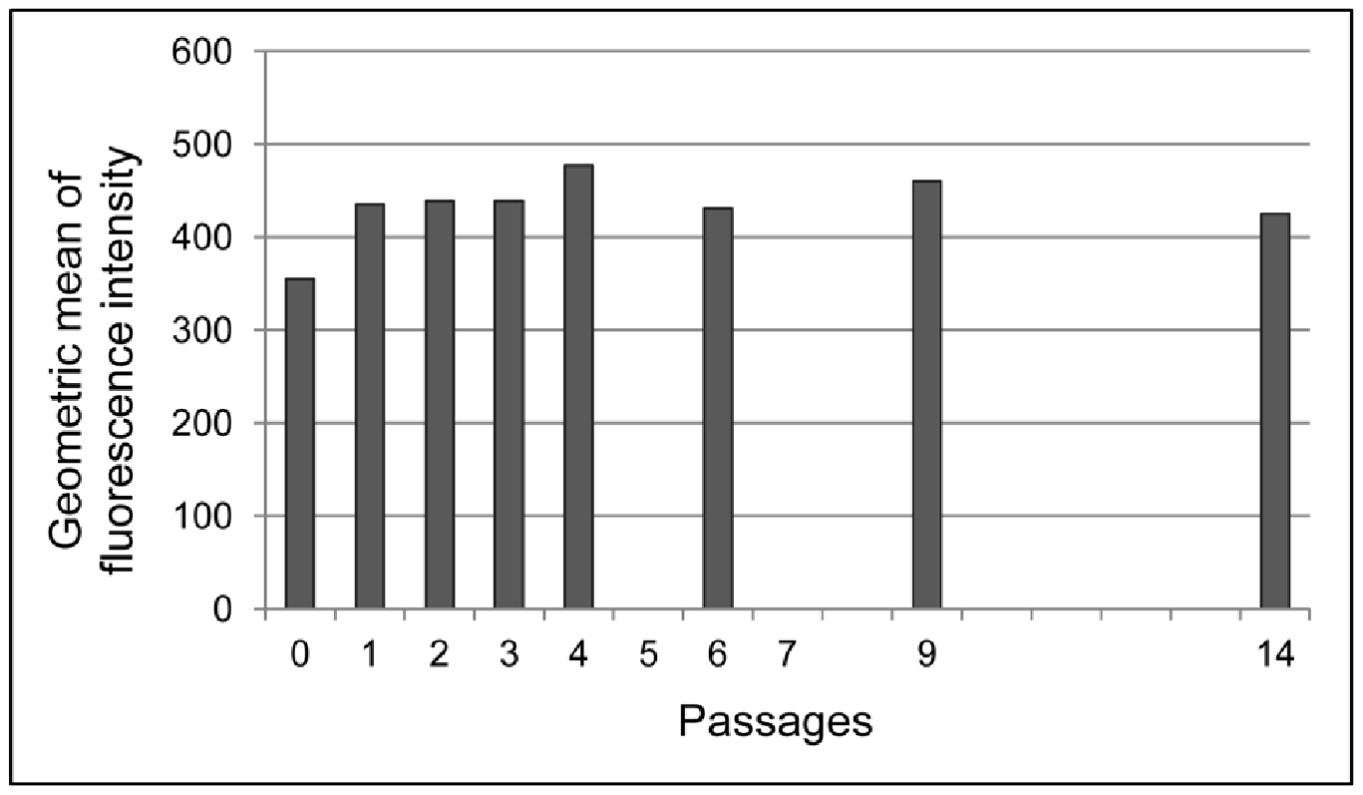
Telomere length dynamics in culture. Geometric mean of fluorescence intensity is reported at different culture passages relative to sample A, as representative example.

Telomere length expressed as mean fluorescence intensity showed an inter-individual variability, independently of donor ages and culture passages. In each subject, ASC showed a low degree of random fluctuation in the telomere dynamics (sample A is reported by way of example, Fig. 5).

A general negative trend during *in vitro* culture was noticed even if no significant telomere length differences were observed within passages from 0 to 6, as determined by the Friedman ANOVA test. Medians and [interquartile ranges] of fluorescence intensity were: 423.5 [381.0–445.0] at P0; 435.0 [414.0–435.0] at P1; and 390.5 [371.5–419.0] at P6.

Relative telomerase activity (RTA) was permanently undetectable in the majority of samples (78.3%). Few samples (17.4%) displayed a marginal telomerase activity (ranging from 0.4 to 78% of the low control provided in the kit), mainly at the beginning of the culture period, within the second passage. Only one sample (sample C at passage 0) showed a little bit higher RTA (248% of the low control), that was no more observed in the following passages. This RTA was however remarkably lower than the activity measured in the K562 tumor cell line used as positive control (%RTA: 5266), therefore these results are considered essentially as negative.

### Soft-agar Growth

A (P14), B (P6), D (P3, P7) F (P0, P2) samples (intentionally tested at passages displaying karyotype alterations) showed no anchorage-independent growth in soft agar after 20 and 30 days, thus indicating no malignant transformation, even when samples from the more advanced passages were cultured (not shown). Conversely, Saos-2 positive control cells grown in the same conditions were able to form colonies (not shown).

## Discussion

Regenerative medicine offers great promise for osteo-cartilagineous medical applications and safety is emerging as critical issue, particularly when *in vitro* cell processing is performed before therapeutic approaches. In Europe, MSCs are considered advanced therapy medicinal products referring to the European GMP rules. Production processes need to ensure not only microbiological safety, but also genetic stability in order to avoid transformation risks. Only few papers have reported transformation in culture of human mesenchymal stem cells [36] and some of these reports have been retracted [37]–[40] because transformation was found to be due to cross-contaminating cancer cells. However, it has been shown that *in vitro* culture induces chromosomal aberrations, which are a hallmark of human cancer. Despite the conflicting results about the neoplastic transformation of human MSC, it should be mentioned that no cases of tumour formation have been documented in human patients after MSC treatment for a variety of indications (over 1000 patients treated until 2012) [1].

Anyway, it appears crucial to perform a detailed analysis of the genome prior to any cell-based treatment. No international indications about genetic stability assessments have been provided and karyotyping is the most frequently used analysis, even if its sensitivity (in the order of Mb) does not allow small molecular change detection; therefore possible genetic abnormalities might be underestimated.

The relevance of the clear tendency towards increased aneuploidy with culture passages is still being debated [33], [35]. It seems that each type of stem cell is prone to acquire specific chromosomal aberrations, and that these aberrations are frequently the same that occur in tumors of the respective tissue [31]. Recurrent aberrations in MSCs may simply imply the acquisition of a growth advantage or they may indicate the beginning of a transformation process in culture [35].

The scientific community would benefit from large scale studies evaluating common genetic changes occurring during *in vitro* culture and their significance to avoid excessively high restrictions to MSC-based promising therapies [57]. However, monitoring of genomic stability should be performed regularly to ensure safe administration and this is particularly crucial for non-life threatening pathologies such as osteoarthritis. The European AdipoA project is a multi-dose phase I clinical trial for therapeutic applications of ASCs in human knee OA and a phase II controlled study. At present, the clinical phase is in progress and the follow up is ongoing. Pre-clinical studies on clinical grade ASC performed by other groups involved in this project suggested a paracrine role of ASC cells in the diseased articulation. Indeed, ASCs were demonstrated to reduce hypertrophy and maintain chondrocyte phenotype in co-culture with primary OA chondrocytes, partly through HGF secretion [58] and COX-2/PGE2 pathway involvement [59]. However, injected ASC seem to persist in vivo in the host as demonstrated in animal models. SCID mice receiving intrarticular injection of human ASCs showed 15% of the injected cells detectable in the joint in the first month and 1.5% engrafted over the long term [60]. In an experimental rabbit model of OA, the short term local bio-distribution of autologous injected ASCs revealed a localization of the cells in the synovium but not in the cartilage, supporting their prevalent trophic effect in inhibiting OA progression [61].

In this study, the impact of *in vitro* culturing of adipose-derived stromal cells on genetic stability has been addressed, which would have an impact on the quality control in cell therapies. In our case series of ASC samples, we observed a proliferation rate slowdown with culture passages, as expected, very similar in all samples except one, showing early deceleration of growth, thus indicating a peculiarity of this donor sample.

No karyotype alterations were observed in culture passages preceding *in vivo* injection (end of P1). Conversely, sex chromosome aneuploidies were detected in three out of six samples, only at advanced PD. The phenomenon of sex chromosome loss has already been described with aging [62]–[66], therefore it might be interpreted as a non-phenotypic event, related both to donor age and culture time. In accordance, the phenomenon was detected only at the most available advanced PD for all the three samples even if no statistical significance was found neither with donor age nor with culture time, probably due to the reduced number of samples. Once again, no autosomal aneuploidies were detected except in sample D at P3 and P7. This donor did not show clinical peculiarities and neither his age nor gender were correlated with the appearance of autosomal abnormalities, therefore we can only speculate about these findings supposing a particular susceptibility of the cells of this donor in culture. In general, as the incidence of aberrations increases with culture passages, and no clear evidence of the role of such alterations was provided, minimizing the culture time is a way to decrease the chance for *in vitro* genetic alterations and to guarantee safety.

Concerning the stability of short repeated sequences in the genome, no cases of MSI in the five analyzed loci were detected, suggesting that genomic stability at short repeated sequences is maintained overtime. In agreement with this, no significant modifications of MMR gene expression, whose activity is required for the stability of short repeated sequences, was noticed by array-analysis. These results differ from those obtained in other cellular models of *in vitro* expansion, where a positive correlation between MSI phenotype and culture extension was observed [48]–[49], [67]–[69]. Moreover, ASCs do not seem to develop the microsatellite instability displayed by human hematopoietic progenitor cells as a function of age [50].

Array-based gene expression analysis for genes involved in DNA damage signaling pathways showed a general down-regulation of a few genes with culture time, namely BRCA1, DMC1, EXO1, FEN1, and GTSE1, which more likely mirrors *in vitro* aging than initial processes towards transformation. Indeed, global changes in mRNA expression profiles of human BM-MSCs cultured to senescence were described with culture time and, in particular, a progressive down-regulation of genes involved in DNA repair and metabolism were observed [70].

BRCA1 is a tumor suppressor gene with a fundamental role in genomic stability maintenance, involved in DNA damage repair, chromatin remodeling and checkpoint activation and BRCA1-defective cells are significantly impaired in the main DNA repair mechanisms [71]–[72]. The down-regulation of this gene might therefore indicate a reduced ability of ASCs at advanced culture passages to respond and correct occurring DNA alterations, with an augmented chance of accumulating DNA damage. DMC1 is a meiosis-specific recombinase, a member of the RecA/Rad51 recombinase superfamily that functions in homologous recombination with the ubiquitously expressed homolog Rad51 gene [73]. The down-regulation of the DMC1 gene probably has no functional effect, as this gene’s activity is only required during meiosis. EXO1 endonuclease has been implicated in a multitude of eukaryotic DNA pathways including repair, recombination, replication and telomere integrity maintenance [74]. It is involved in mutation avoidance pathways both MMR-dependent and independent. As for BRCA1, its down-regulation might increase the mutation rate. FEN1 is a multifunctional nuclease, a member of the Rad2 family, participating in distinct DNA metabolic pathways including DNA replication and repair, telomere maintenance and apoptotic DNA fragmentation, whose inactivation results in genomic instability [75]. GTSE1 protein is almost undetectable in the G1 phase, whereas it increases during S and peaks in the G2 phase of the cell cycle. In response to DNA damage it accumulates in the nucleus and binds p53 repressing its ability to induce apoptosis [76]. The observed reduced expression of its mRNA might reflect the reduced percentage of dividing cells at advanced culture times.

Sample D, showing autosomal aneuploidies, also displayed a different gene expression pattern at advanced PD with few genes, all upregulated: BTG2, involved in cell cycle regulation with anti-proliferative activity [77]; GADD45G, whose transcript levels are increased following stressful growth arrest conditions, participating in cell cycle arrest, DNA repair, cell survival and apoptosis in response to environmental and physiological stress, as well as having a role in development and carcinogenesis [78]; IP6K3 phosphokinase and MAP2K6, dual specificity protein kinases that activate p38 MAP kinase, involved in apoptosis, stress response and transcriptional regulation [79]. Conclusions about these data can only be speculative, since they came from only one sample, but they might be correlated with the different behavior of this donor sample compared to that of the others in terms of replication rate slowdown and chromosomal stability, thus suggesting a patient-dependent profile.

We also observed that ASCs lack detectable telomerase activity in agreement with other reports [80]–[81], thus suggesting that these cells are not prone to transformation *in vitro*. Given this observation, concomitant telomere erosion was expected; however, only a slight trend of telomere attrition over culture time was observed, which was less evident than that revealed, by densitometric analysis, in few ASC samples from various anatomical sites [82]. Although ASCs were observed for several passages, cumulative evaluations were performed until P6, when the growth curves were still nearly linear and before the pre-senescent/senescent phase, which is expected between P10 and P20, as reported by others [21]. However, it cannot be excluded that, as described in synchronized cultures of bone marrow mesenchymal stem cells [83], also the present ASC populations display telomerase activity during the S phase, but this activity is hidden in the present asynchronous culture system.

Overall, the present results argue for the absence of genetic damage accumulation at the first culture passages and their occasional appearance at advanced passages only in specific cases, thus pointing to the importance of reducing culture times as much as possible. It is however noteworthy that even samples showing chromosomal alterations did not display anchorage-independent growth, in favor of the hypothesis that these changes do not foster a selective growth advantage *in vitro* to abnormal cells.

## Conclusions

A panel of analyses were performed including both cytogenetic and molecular DNA damage evaluation showing that, after repeated duplications *in vitro* in defined culture conditions, human adipose stromal cells do not show alterations in either chromosome or short repeated sequences, do not show telomere attrition, do not express significant amounts of active telomerase and do not acquire anchorage-independent growth ability, and all data support their safety for therapeutic use.
